# Separation and Recovery of Trace Silver from Sintering Filtrated Dust of Ferrous Metallurgy via Complexation Leaching

**DOI:** 10.3390/molecules30061339

**Published:** 2025-03-17

**Authors:** Zhiqiang Qiao, Yunquan Yang, Tian Zhang, Weishun Chen

**Affiliations:** 1 School of Chemical Engineering, Xiangtan University, Xiangtan 411105, China; 202031000181@smail.xtu.edu.cn (Z.Q.);; 2 School of Chemical Engineering, Zhaoqing Technician Institute, Zhaoqing 526040, China; chenweishun3@163.com

**Keywords:** sintering filtrated dust, Na_2_S_2_O_3_-CuCl system, leaching, silver recovery, hydrogen peroxide purification mechanism

## Abstract

In this work, by using the sintering filtrated dust (SFD) from ferrous metallurgy as a raw material, the process of separating and recovering trace silver, including the steps of complexation leaching by Na_2_S_2_O_3_-CuCl, purification by hydrogen peroxide, and precipitation transformation, was researched and developed. The process is characterized by a high leaching selectivity and a high recovery. The recommended conditions for leaching trace silver from SFD were as follows: a leaching time of 120 min, a leaching temperature of 60 °C, a solid–liquid ratio of 6 L/kg, a Na_2_S_2_O_3_ concentration of 45 g/L, and a CuCl dosage of 5.0 g/L. Through a two-step hydrogen peroxide process, removal of the impurity ions Cu and Pb and high-efficiency recovery of trace silver were realized. The purity of the silver sulfide product obtained from the recovery was 97.0%, and the total silver recovery was 80.1%. In addition, the reaction mechanism of the recovery process was investigated in this work, and effective removal of impurity ions was realized by regulating the reaction time.

## 1. Introduction

Owing to its unique physicochemical properties, such as excellent corrosion resistance, excellent electrical conductivity, and catalytic activity, silver is widely used in electronics and industrial catalysis. For example, silver is often used in the electronics industry to manufacture high-performance components such as circuit boards, conductive pastes, and solar cells [1,2]. In industrial catalysis, silver is widely used as an important catalyst that can effectively promote reactions such as selective oxidation, hydrogenation, and methanation in chemical production [3,4]. In addition, the potential applications of silver-based catalysts in new energy and environmental protection, especially in electric vehicles and clean energy conversion technologies [5,6,7,8,9,10], have attracted much attention.

However, the scarcity of global silver resources poses a significant challenge to the supply chain. According to the World Silver Institute, the global silver demand in 2023 reached 35,551 tons, whereas the supply was only 31,100 tons, resulting in a supply–demand gap of approximately 4400 tons. The supply–demand gap is expected to widen to approximately 5000 tons by 2024, although the production of mineral silver is expected to recover, leading to a 2–3% increase in supply, i.e., to 31,700 tons. Thus, the recovery of scrap silver, especially from industrial waste, is becoming increasingly important and will play a key role in the future silver supply.

During steel metallurgy, silver enters the smelting process with iron ore and eventually concentrates in sintering ash. Although the silver content in SFD is only approximately 100 g/t, considering the large amount of global crude steel production, which reached 1.83 billion tons in 2022, the production of SFD accounts for 1.5–2.0% of the total crude steel production, which means that the amount of silver that can be recovered from SFD is very considerable. Therefore, the recovery of silver resources from SFD has important economic and environmental value.

Currently, hydrometallurgy is the most commonly used technique for the extraction and recovery of silver from silver-based scraps, with chloride leaching being the dominant method because of its lower toxicity and cost [11,12,13,14]. In this method, silver is dissolved by a chloride solution and recovered through a precipitation or electrodeposition process [15,16,17,18,19]. Despite the advantages of the chloride leaching method, it still requires high-temperature and strongly acidic conditions, which greatly increases the operational difficulty and environmental burden. Therefore, the development of more environmentally friendly and simpler recovery processes has become one of the hotspots in current research.

In recent years, the thiosulfate method has been widely studied as a green alternative technology, especially in the field of gold recovery, with remarkable results, and has been gradually applied to silver recovery [20,21]. In this method, divalent copper is used as a catalyst, and ammonia is used as a complexing agent to enable thiosulfate to form stable complexes with gold and silver. However, the use of ammonia may produce an irritating odor and pollute the environment, while the complexation reaction system increases the difficulty of the process.

To solve these problems, a novel process based on the Na_2_S_2_O_3_-CuCl system for silver recovery from iron and steel metallurgical sintering ash is proposed in this paper. The process does not require the addition of ammonia, thus avoiding the environmental pollution caused by ammonia, and the silver leaching process is optimized by gradually adding hydrogen peroxide, which effectively removes impurity ions such as copper and lead. In this work, the effects of different single factors (e.g., temperature, thiosulfate concentration, and reaction time) on the leaching process were systematically investigated, and ultimately, a high-purity silver sulfide product was obtained by optimizing the process conditions. This new process not only is easy to operate and environmentally friendly but also significantly improves the silver recovery efficiency, providing a new technological pathway for the recovery of silver from industrial wastes, which is highly important for the realization of the sustainable use of resources.

## 2. Results and Discussion

Since the SFD contains a large amount of water-soluble compounds such as KCl and NaCl, to further enrich the target metal elements and reduce the amount of water-soluble impurity metal elements in the leaching solution, the SFD needs to be pretreated by being washed with water before the leaching experiment. The water washing pretreatment steps were as follows: 50.0 g of sintering ash (dry basis) was added to 200 mL of deionized water, stirred in a 50 °C water bath with a mixer at 400 rpm for 45 min, and filtered under a vacuum. The filter cake was rinsed with deionized water for 5 min after filtration to remove soluble alkali compounds on the surface of the cake. Afterward, the filter cake was dried in an oven at 105 °C for 10 h and crushed.

The leaching process was conducted by adding 50.0 g of pretreated SFD to 300 mL of a 45 g/L Na_2_S_2_O_3_ solution containing 0.05 mol/L CuCl. The mixture was stirred at 60 °C for 120 min, filtered, and treated with hydrogen peroxide in two stages to remove impurities (Cu, Pb) and recover silver.

The physicochemical properties of SFD and its derivatives—including raw materials, washed ash, leaching residues, intermediates, and final products—were analyzed using X-ray diffraction (XRD), atomic absorption spectroscopy (AAS), laser particle size analysis, and Fourier-transform infrared spectroscopy (FTIR). These methods assessed phase composition, elemental distribution, particle morphology, and functional group variations across different processing stages.

### 2.1. Composition of SFD

The main metal elements of SFD before and after pretreatment were determined via a polarized Saiman atomic spectrophotometer, and the contents of several of the main metal elements were obtained, as shown in Table 1.

The data in Table 1 show that the SFD contains high contents of Fe and K, as well as small amounts of Na and Pb and trace amounts of Cu and Ag, which is consistent with the XRD analysis results.

**Figure 1:**
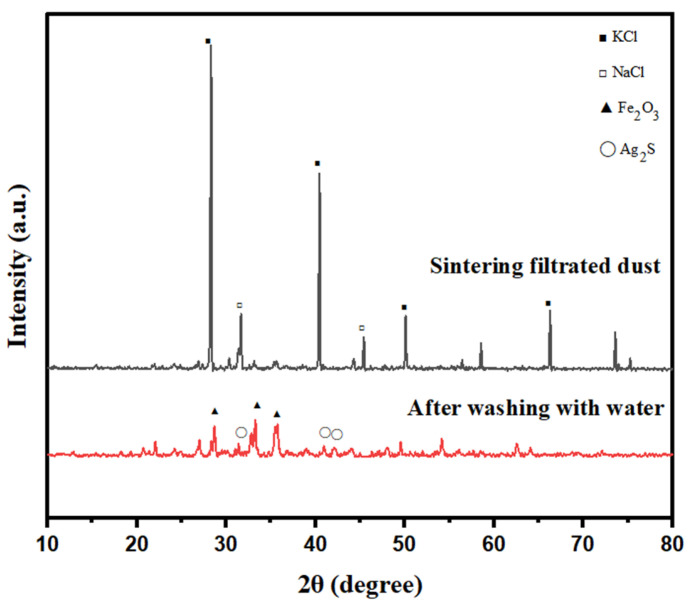
Physical phase changes in the SFD after pretreatment.

The main physical phases of SFD before and after pretreatment were characterized and analyzed using an X-ray polycrystalline powder diffractometer (XRD), and the results are shown in Figure 1. Figure 1 shows that the diffraction peak with the largest intensity in the XRD pattern of the SFD material is the characteristic peak of KCl, whereas other characteristic peaks are not observed, probably because of the high content of potassium in the SFD, which absorbs a large amount of X-rays during the measurement. Other elements show weak absorption, resulting in the KCl peak being too intense and masking the other characteristic peaks.

The characteristic peak signals of KCl and NaCl in the XRD pattern of the pretreated SFD are obviously weaker because KCl and NaCl are extremely soluble in water, and most of the KCl in the SFD has been removed after washing with water. At the same time, the characteristic peaks of substances such as Fe_2_O_3_, PbO, CuO, and Ag_2_S appear in the spectrum, which indicates that the other main phases in the SFD are Fe_2_O_3_, PbO, CuO, and Ag_2_S.

Water washing removed 62.6% of the initial SFD mass (from 50.0 g to 18.7 g), primarily through dissolution of alkali salts. This mass reduction concentrated insoluble phases, increasing the relative silver content from 160 g/t to 500 g/t. However, absolute silver retention was 116.9%, suggesting partial loss via dissolution or ultrafine particle entrainment. The interplay between mass loss and concentration effects underscores the need to optimize washing parameters for critical metal recovery.

### 2.2. Particle Size Analysis of SFD

**Figure 2:**
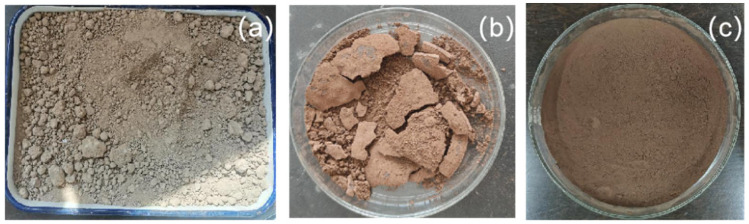
SFD morphology under different treatments: (**a**) Raw SFD; (**b**) SFD after water washing; (**c**) SFD after sieving.

Figure 2a,b below show the SFD before and after washing, respectively, and Figure 2c shows the SFD after sieving.

**Figure 3:**
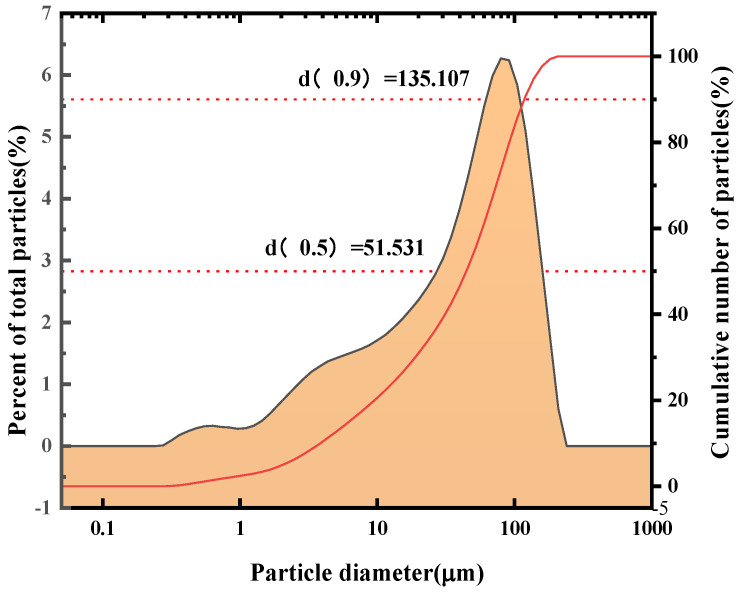
Particle size of SFD after washing with water.

The sieved SFD was subjected to particle size analysis, and the results are shown in Figure 3. The test was carried out with anhydrous ethanol as the dispersing medium and a Mastersizer 2000 laser particle sizer, and the characteristic parameters are shown in Table 2.

As shown in Figure 3, the distribution range of the SFD particle diameters after washing and sieving is more concentrated, with more than 90% below 135 μm. The difference between the median diameter and the volume average particle size is large, and the distribution of the sample particle sizes is asymmetric. The difference between the volume average particle size and the surface area average particle size is large, indicating that the particle shape is irregular and that the particle size distribution is not concentrated.

Overall, the SFD particles after sieving have a larger specific surface area and a smaller particle size, so they can fully contact the leaching solution, which is favorable for the subsequent leaching recovery. Moreover, after washing with water and sieving, the elements in the SFD are more evenly distributed, which is favorable for the subsequent leaching effect.

### 2.3. Removal of Potassium from SFD

**Figure 4:**
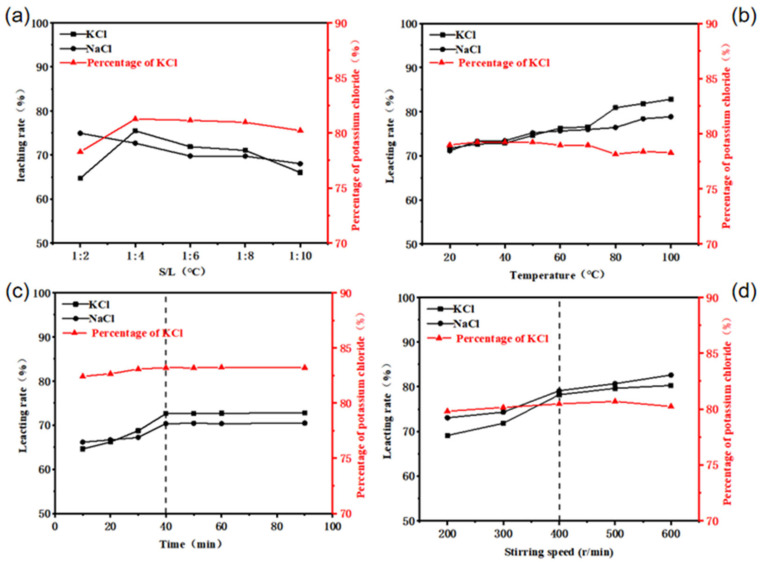
Influence of various factors on the effect of KCl removal from SFD. (**a**) Solid–liquid ratio. (**b**) Temperature. (**c**) Time. (**d**) Stirring speed.

The removal efficiency of metallic elements from SFD is influenced by multiple variables, as demonstrated in prior studies on solvent crystallization processes [22,23,24]. To systematically optimize KCl extraction, this work investigated critical operational parameters including solid–liquid ratio, temperature, agitation time (reaction duration), and stirring speed, with results comprehensively summarized in Figure 4. Building upon the phase equilibrium principles established in our earlier quaternary system research [24], the optimized conditions were determined as follows: a solid–liquid ratio of 1:4 g/mL, ambient temperature (25 °C ± 2 °C), a reaction duration of 40 min, a stirring speed of 400 rpm, and 0.08 g sodium dodecylbenzene sulfonate (SDBS) dispersant per 100 g SFD. These parameters align with the kinetic correlations reported for similar metallurgical dust systems [23]. Under these conditions, the removal efficiencies of KCl and NaCl reached 93.9% and 85.9%, respectively, demonstrating significant consistency with theoretical predictions from our previous models [22,24].

### 2.4. Na_2_S_2_O_3_-CuCl Leaching of SFD

**Figure 5:**
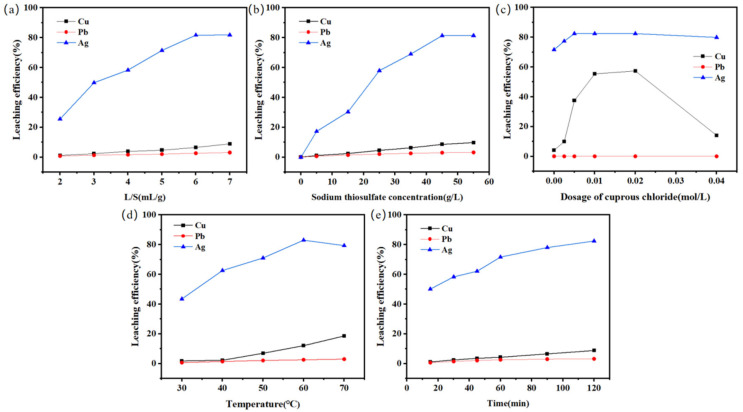
Influence of various factors on the leaching efficiency. (**a**) Liquid–solid ratio. (**b**) Sodium thiosulfate dosage. (**c**) CuCl dosage. (**d**) Temperature. (**e**) Time.

The effects of the liquid–solid ratio (L/S), sodium thiosulfate dosage, CuCl dosage, temperature, and time on the leaching efficiency of several metal elements in SFD were investigated, and the results are shown in Figure 5. The recommended leaching conditions are a sodium thiosulfate concentration of 45 g/L, an L/S of 6 mL/g, a leaching temperature of 60 °C, a cuprous chloride dosage of 0.005 mol/L, and a leaching time of 120 min, and the Ag, Cu, and Pb leaching efficiencies are 83.1%, 8.77%, and 3.16%, respectively, under these conditions.

### 2.5. Removal of Copper and Lead from the Leaching Solution

**Figure 6:**
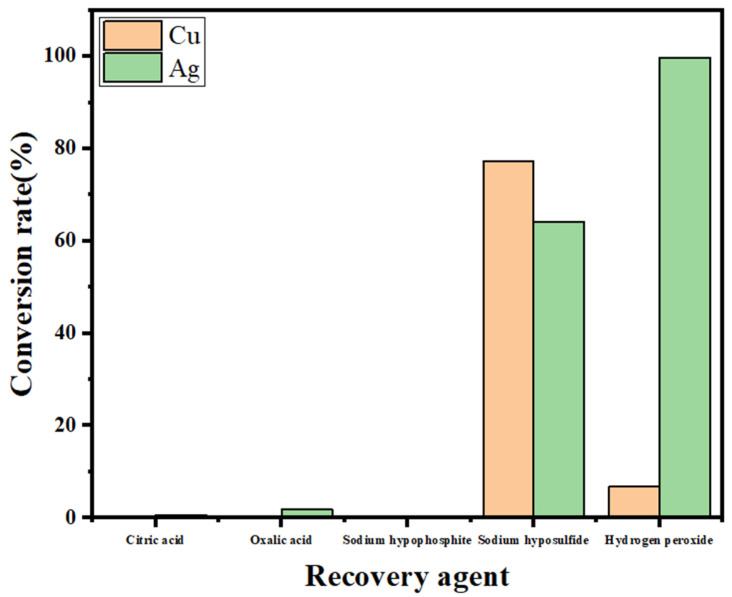
Effects of different recovery agents on the separation effect.

The leaching solution obtained from leaching of SFD by the thiosulfate-cuprous chloride system contains Ag, Cu, and Pb. Due to the low content of silver ions in the solution, adjustment of the pH and replacement methods cannot achieve selective separation of impurity metals, resulting in the loss of a large amount of silver. To achieve impurity metal (Cu, Pb) removal and silver recovery, citric acid, oxalic acid, sodium hypophosphite, sodium hypophosphite, sodium hypophosphite, and sodium dithionite were experimentally compared. Hydrogen peroxide was used as a separating agent for the separation and recovery effects, and the results are shown in Figure 6.

As shown in Figure 6, the conversion rates of citric acid, oxalic acid, sodium hypophosphite and hydrogen peroxide to copper and silver are very low, whereas the conversion rates of sodium dithionite to copper and silver are 77.35% and 64.23%, respectively, which are high conversion efficiencies, but they cannot be separated. The conversion rate of hydrogen peroxide to silver reaches more than 99%, whereas the conversion rate for the impurity metal copper is only 6.74%, which can achieve preliminary separation. Therefore, hydrogen peroxide was chosen as the precipitation conversion agent for selective recovery of silver. According to experiments, when the reaction time is short and the hydrogen peroxide concentration is low, copper and lead will react with hydrogen peroxide first to produce a precipitate, while the conversion rate of silver is very low. Therefore, this characteristic was fully exploited to carry out stepwise addition of hydrogen peroxide for separation and recovery of silver.

**Figure 7:**
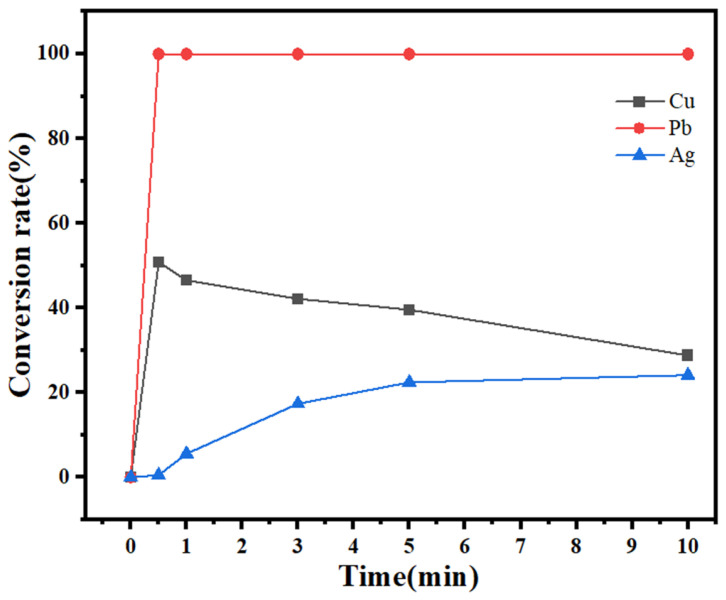
Effect of the reaction time on the conversion rate.

**Figure 8:**
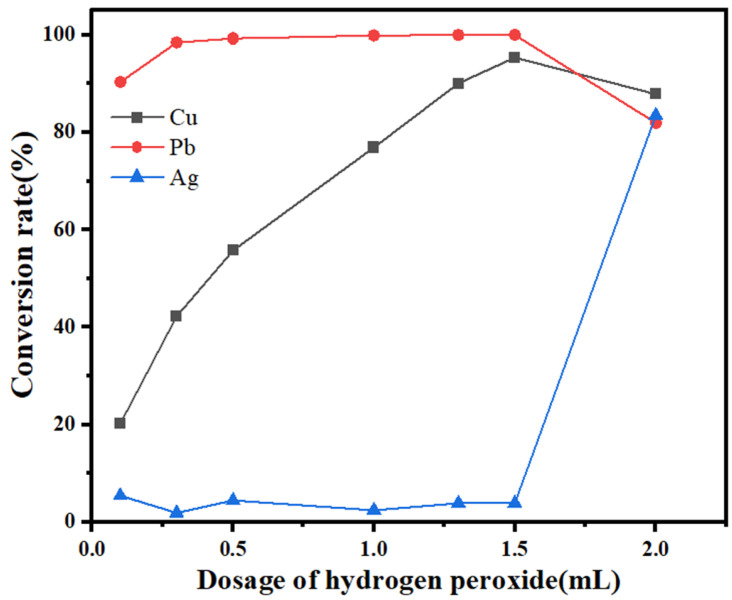
Effect of the hydrogen peroxide dosage on the conversion rate.

To maximize the removal of copper and lead and the enrichment of silver, the effects of different hydrogen peroxide dosages and reaction times on the conversion of metal ions in the leaching solution were investigated, and the results are shown in Figure 7 and Figure 8.

At 30 °C, 100 mL of leaching solution was taken, 1 mL of hydrogen peroxide was added, and the stirring speed was 100 rpm. The copper and lead conversion rates peak at 0.5 min. With a further extension of the reaction time, the copper conversion rate gradually decreases, whereas the lead conversion rate basically remains unchanged. The silver conversion rate increases with increasing reaction time and stabilizes after 5 min. To obtain good separation, 0.5 min was chosen as the recommended reaction time.

Hydrogen peroxide (30%) was added to 100 mL of leaching solution at 30 °C, with a stirring speed of 300 rpm and a reaction time of 5 min. When the dosage of hydrogen peroxide is 0.3 mL, the lead conversion rate reaches 100%, the copper conversion rate is 40%, and the silver conversion rate is only approximately 5%. With a gradual increase in the hydrogen peroxide dosage, the lead conversion rate remains stable, the copper conversion rate gradually increases, and the silver conversion rate does not change much. When the hydrogen peroxide dosage reaches 1.5 mL, the copper conversion rate reaches more than 95%, whereas the silver conversion rate is still approximately 5%. When more hydrogen peroxide is added, the conversion products of copper and lead dissolve, and the conversion rates decrease, whereas the silver conversion rate increases to 83%. To maximize the separation of copper, lead, and silver, the amount of hydrogen peroxide was chosen as 1.5 mL, at which the lead conversion is 100%, the copper conversion is 95%, and the silver loss is 5%.

### 2.6. Separation and Recovery of Silver from Purified Liquids

**Figure 9:**
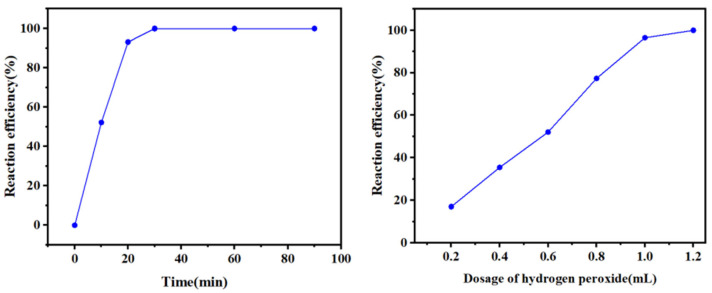
Effects of the reaction time and hydrogen peroxide dosage on the reaction efficiency.

The purified solution was obtained after impurity removal. Hydrogen peroxide was added to separate and recover silver, with a reaction temperature of 30 °C, a stirring speed of 300 rpm, and a purified solution amount of 100 mL. The effects of the amount of hydrogen peroxide and the reaction time on silver recovery were examined, and the results are shown in Figure 9.

As shown in Figure 9, 100% silver recovery is achieved at a dosage of 1.2 mL of H_2_O_2_ (30%)/100 mL of purification solution, a reaction time of 30 min, a reaction temperature of 30 °C, and a stirring speed of 300 rpm.

**Figure 10:**
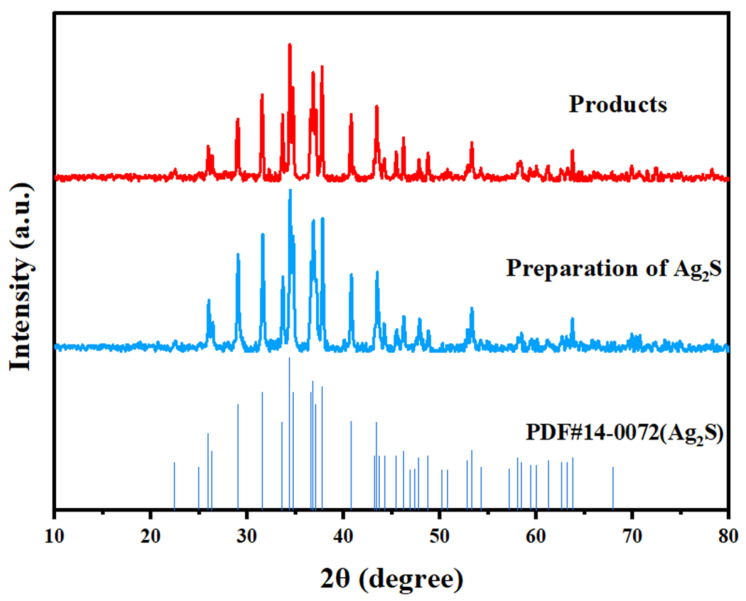
XRD patterns of the isolated recovered products.

The products recovered from hydrogen peroxide separation were analyzed via XRD, and the results are shown in Figure 10.

As shown in Figure 10, the characteristic peaks of the products at 2θ = 28.98°, 31.52°, 34.38°, 36.81°, 40.74°, and 43.41° basically correspond to the standard profile of Ag_2_S. These peaks are essentially the same as those of the pure substance Ag_2_S, as verified by comparison, indicating that the obtained product is Ag_2_S. The purity of the obtained product Ag_2_S was determined. The average value of the Ag_2_S content in the product is 97.2%, which means that the Ag_2_S purity is 97.2%.

## 3. Analysis of the Reaction Mechanism in the Recovery Process

### 3.1. Kinetics of Sodium Thiosulfate Extraction

The reaction kinetics, which are the nonequilibrium dynamics that vary with time, can be used to investigate changes in the efficiency and the chemical reaction mechanisms during reactions, and different models are applied for different types of reactions [25,26,27]. The leaching of silver from SFD by the thiosulfate system is a typical liquid-solid nonhomogeneous reaction, and although the SFD composition is complex, the process is simplified by modeling [28]. The leached SFD has a uniform particle size distribution and can be regarded as consisting of spherical particles. As the reaction proceeds, the part of the particles in contact with the solution gradually dissolves, and the unreacted nuclei gradually shrink at a uniform efficiency [29]; thus, the model of shrinking nuclei in a liquid-solid inhomogeneous phase reaction is suitable [30,31].

The leaching process can be divided into five steps: the leaching agent passes through the surface liquid film of the solid particles (external diffusion); the leaching agent passes through the solid particles to the reaction interface (internal diffusion); the leaching agent undergoes a chemical reaction at the reaction interface, followed by the reaction products passing through the solid particles to the liquid film (internal diffusion); and the reaction products pass through the surface liquid film of the solid particles (external diffusion) [32]. The condensation reaction model in the leaching kinetic model fits well with this leaching process. Each of the control types corresponds to a different equation.

For chemical reaction and external diffusion control,

(1) $$1-{\left(1-\omega \right)}^{\frac{1}{3}}=k_{1}t$$

where ω is the silver leaching efficiency and k_1_ is the chemical reaction and external diffusion efficiency constant.

The activation energy controlled by chemical reactions is large, typically 40–300 kJ/mol, whereas the external diffusion-controlled activation energy is approximately 8–20 kJ/mol.

When the reaction is internal diffusion controlled,

(2) $$1-\frac{2}{3}\omega {-(1-\omega )}^{\frac{2}{3}}=k_{2}t$$

**Figure 11:**
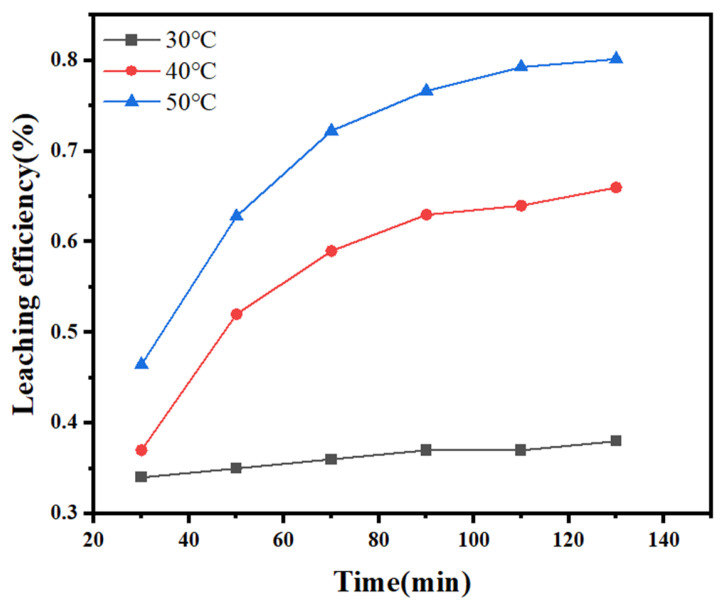
Effect of the temperature on the silver leaching efficiency.

The leaching efficiencies over time at a sodium thiosulfate concentration of 25 g/L, an L/S of 6 mL/g, and a temperature of 30, 40, and 50 °C were determined, as shown in Figure 11.

As shown in Figure 11, the silver leaching efficiency in the thiosulfate solution increases with increasing temperature. Under low-temperature conditions, the conversion rate slightly changes with time. In contrast, at higher temperatures, the conversion is rapid, gradually levels off with time, and basically stabilizes at approximately 130 min.

**Figure 12:**
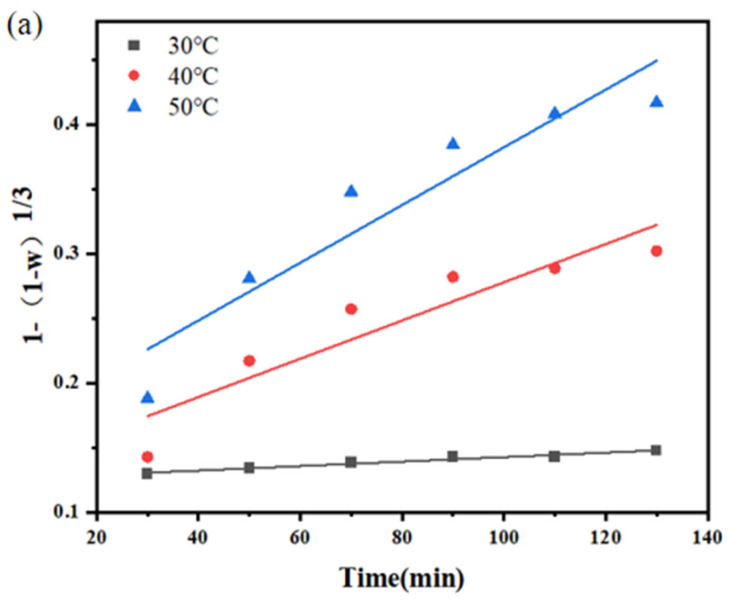

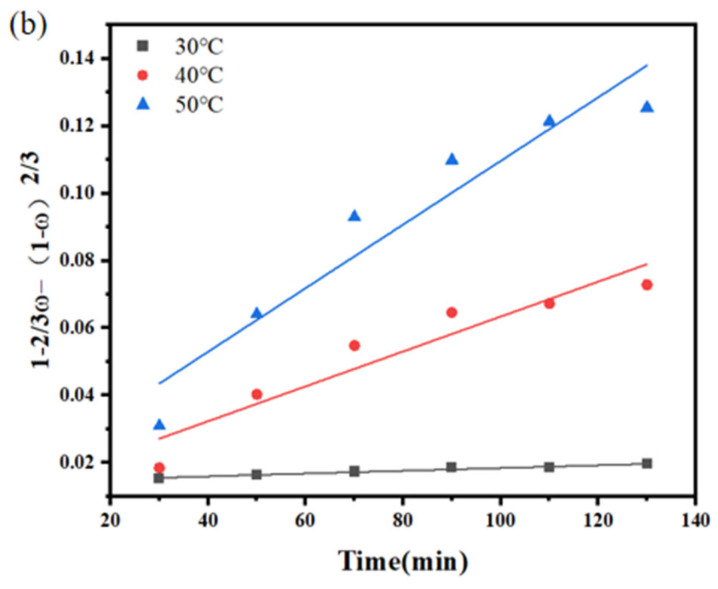
Results of fitting different diffusion efficiency equations: (**a**) chemical reaction and external diffusion efficiency equations; (**b**) internal diffusion efficiency equations.

To understand the control steps of this leaching process, the leaching efficiencies obtained at different temperatures were substituted into the calculation equations to obtain the k values under different controls, as shown in Figure 12. The fitting results are shown in Table 3 and Table 4 below.

The kinetic model fitting results (Table 3 and Table 4) show comparable R^2^ values for both chemical reaction/external diffusion (0.86–0.96) and internal diffusion (0.90–0.97) controls, with no statistically significant preference for either model. Levenspiel explicitly states that R^2^ > 0.9 applies to single-mechanism systems (pure chemical or diffusion control) [33]. In our study, the ORP fluctuations (0.15–0.3 mV) indicate a transition region where chemical oxidation (S_2_O_3_^2−^ bond cleavage) and diffusion (mass transfer of reactants) co-dominate. This aligns with Székely et al., who showed that mixed control inherently reduces R^2^ due to competing rate-determining steps [34].

Experimental validations through particle size reduction and agitation tests confirm that external mass transfer has minimal influence, while the partial dependency on particle size suggests transient internal diffusion effects in coarse particles. Thus, the overall process is predominantly governed by surface chemical reactions, with internal diffusion contributing only during initial stages.

The leaching process is best described by a hybrid mechanism: initial rapid dissolution is mildly hindered by internal diffusion in larger particles (d50 > 45 μm), while the overall rate is governed by chemical reactions at the mineral–solution interface. This interpretation reconciles the intermediate Ea value, moderate particle size sensitivity, and superior MAE of the chemical reaction model. The negligible impact of agitation speed further excludes external diffusion as a significant factor.

**Figure 13:**
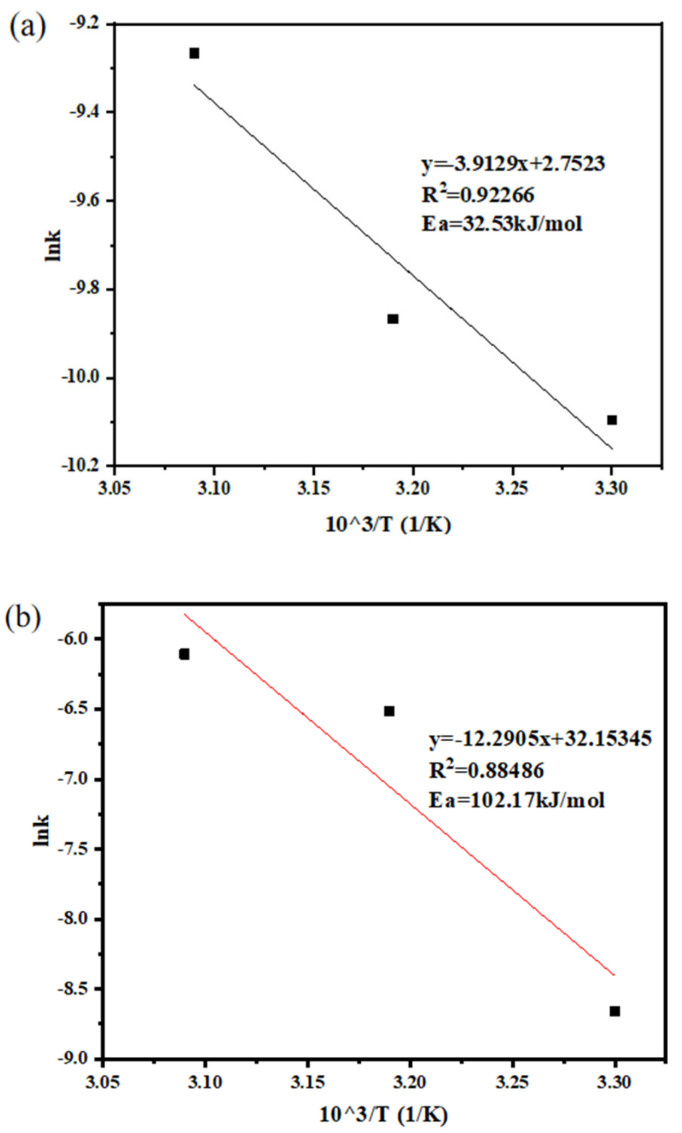
(**a**) Arrhenius equation fit for chemical reaction and external diffusion control. (**b**) Arrhenius equation fit for internal diffusion control.

The Arrhenius equation was separately fitted, as shown in Figure 13. Among the fitting results, those of the chemical reaction and external diffusion-controlled activation energy are within the appropriate range, and their fitting effect is better; thus, the preliminary judgment is that the control mechanism is chemical reaction and external diffusion control. Roldán-Contreras et al. reported (Ea = 36 kJ/mol) for gold leaching in an ammonia-thiosulfate system [35], closely matching our (Ea = 32.53 kJ/mol). Alvarez et al. reviewed nine recent studies and confirmed (Ea = 28–42 kJ/mol) as the chemical control signature for thiosulfate oxidation [36], aligning with our results.

The current research on thiosulfate leaching systems mainly focuses on the copper–ammonia–thiosulfate system, whereas in the system of this study, copper salt is added in the substrate, and no ammonia is added as a stabilizer; thus, copper-ammonia complexes that can participate in the reaction will not be formed [37]. Therefore, the mechanism of the copper–ammonia–thiosulfate system cannot be directly applied, and possible reaction processes are proposed based on the relevant literature and experimental results, as shown in reaction Equations (3)–(8):

(3) $${\mathrm{CuCl}+S}_{2}{O_{3}}^{2-}\to {\mathrm{CuS}}_{2}O_{3}{+\mathrm{Cl}}^{-}$$

(4) $${\mathrm{CuCl}+2S}_{2}{O_{3}}^{2-}\to {\mathrm{Cu}(S}_{2}O_{3}{{)}_{2}}^{3-}{+\mathrm{Cl}}^{-}$$

(5) $${\mathrm{Ag}}_{2}{S+2\mathrm{CuS}}_{2}{O_{3}}^{-}\to {2\mathrm{AgS}}_{2}{O_{3}}^{-}{+\mathrm{Cu}}_{2}S$$

(6) $${\mathrm{Ag}}_{2}{S+2\mathrm{Cu}(S}_{2}O_{3}{{)}_{2}}^{3-}\to {2\mathrm{Ag}(S}_{2}O_{3}{{)}_{2}}^{3-}{+\mathrm{Cu}}_{2}S$$

(7) $${\mathrm{Ag}}_{2}{S+4\mathrm{Cl}}^{-}\to {{2\mathrm{AgCl}}_{2}}^{-}{+S}^{2-}$$

(8) $${{\mathrm{AgCl}}_{2}}^{-}{+2S}_{2}{O_{3}}^{2-}\to {\mathrm{Ag}(S}_{2}O_{3}{{)}_{2}}^{3-}{+2\mathrm{Cl}}^{-}$$

The thermodynamic stability of the thiosulfate complexes was quantitatively assessed through equilibrium constant analysis. The calculated ln(K) values for Cu(S_2_O_3_)_2_^3−^(ln(K) = 12.5) and Ag(S_2_O_3_)_2_^3−^(ln(K) = 15.2) reveal that silver-thiosulfate complexes exhibit significantly higher thermodynamic stability. This explains the selective transfer of Ag^+^ into solution via ligand substitution, despite the presence of competing copper species. The dominance of silver complexation is further supported by the chloride-mediated pathway, where intermediate AgCl^2−^ rapidly reacts with thiosulfate to form stable Ag(S_2_O_3_)_2_^3−^.

Cuprous chloride is insoluble in water but reacts with thiosulfate to form cuprous thiosulfate complexes. Copper thiosulfate replaces silver in silver sulfide and is converted to cuprous sulfide with bound sulfur ions [37]. Moreover, since cuprous chloride carries chloride ions and potassium chloride and sodium chloride residues exist in the SFD, there may also be chlorine ligands of silver and their conversion with thiosulfate complexation, which would facilitate the reaction. This conjecture is supported by the partial elution of silver during the previous aqueous washing process and the effects of the chloride salt method [38].

Other metals in SFD can also react with thiosulfate to form thiosulfate complexes, depleting the thiosulfate in the solution and inhibiting the silver leaching process. Moreover, thiosulfate itself is substable, and thiosulfate transformation processes may occur in the solution [39].

### 3.2. Mechanism of the Removal of Copper and Lead Impurities from the Leaching Solution

**Figure 14:**
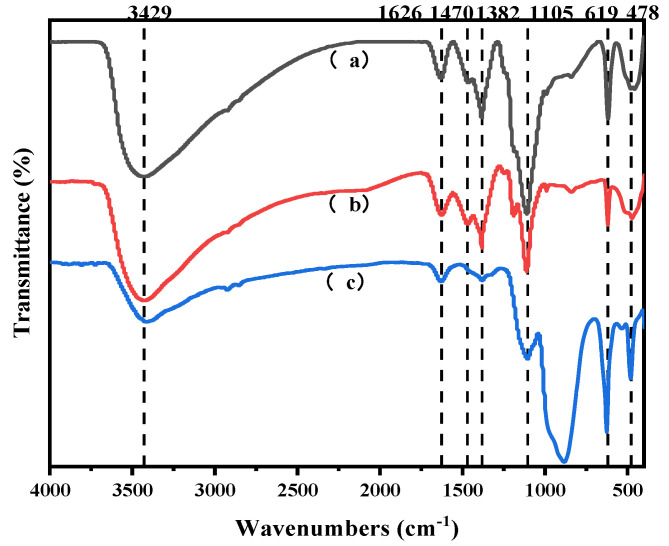
Infrared spectra of intermediates: (a) reaction products of the mixture with hydrogen peroxide; (b) reaction products of copper thiosulfate with hydrogen peroxide; (c) reaction products of lead thiosulfate with hydrogen peroxide.

During the reaction of the leaching solution with hydrogen peroxide, a rapid color change to brownish-yellow was observed, accompanied by gas bubble formation and aggregation of a brown flocculent product. To identify the composition of this product, the solid was treated with dilute HCl. The rapid dissolution of the solid with vigorous gas release and the subsequent light blue coloration of the solution suggested the presence of copper peroxide (CuO_2_) intermediates. This hypothesis was further supported by infrared (IR) analysis of the reaction products (Figure 14a), which revealed characteristic peaks at 1382 cm^−1^ and 1470 cm^−1^, corresponding to CuO_2_ [40].

To clarify the role of copper in the rapid removal of lead, controlled experiments were conducted. In the lead thiosulfate solution (without copper), the addition of H_2_O_2_ resulted in minimal reaction within the first 3 min, with only 20% lead conversion after 5 min, and no gas bubbles were observed. In contrast, immediate gas evolution occurred in the copper-containing thiosulfate solution, indicating the generation of reactive intermediates such as CuO_2_. This stark contrast highlighted copper’s catalytic role in accelerating lead removal. In the leaching solution, lead was completely converted within minutes, likely due to copper-mediated generation of sulfate radicals (SO_4_⋅^−^), which oxidize thiosulfate-bound lead to insoluble lead sulfate (PbSO_4_).

(9) $${\mathrm{Pb}(S}_{2}O_{3}{{)}_{2}}^{3-}{+\mathrm{SO}}_{4}\cdot^{-}\to {\mathrm{PbSO}}_{4}↓{+2S}_{2}O_{3}\cdot^{-}$$

IR spectra of the lead-containing product (Figure 14c) confirmed PbSO_4_ formation through peaks at 619 cm^−1^, 882 cm^−1^, and 1105 cm^−1^ [41,42].

The rapid initial step (CuO_2_ formation) and slower subsequent dissolution–conversion explain the observed decrease in copper conversion over time. By controlling the reaction duration, intermediates like CuO_2_ and PbSO_4_ can be selectively separated, optimizing impurity removal.

## 4. Materials and Methods

### 4.1. Main Instruments and Reagents

Table 5 and Table 6 show the main specifications of the apparatus and reagents used in the investigation, respectively, including their manufacturer.

### 4.2. Experimental Methods

#### 4.2.1. Chemical Composition and Physical Analysis of SFD

(1) Physical phase analysis. The physical phases of the experimental raw materials, i.e., iron and steel metallurgical sintering ash, sintering ash after washing, filter residue after leaching, intermediate products, and final products, were detected and analyzed via a D/max 2550 X-ray diffractometer produced by the Nippon Mechanics Company. The test conditions were as follows: rotating anode, 18 kW; phototube voltage, 40 kV; phototube current, 300 mA; scanning angle range, 5–90°; step size, 0.02°; and scanning speed, 14°/min. The X-ray diffraction (XRD) patterns were analyzed via Jade5.0 software.
(2) Elemental analysis. A total of 0.1000 g of SFD was accurately weighed, completely covered with 2 g of sodium hydroxide, and calcined in a nickel crucible at 700 °C for 2 h. Then, the solid in the crucible was dissolved with 4 mol/L hydrochloric acid and filtered, and the solution was fixed to 100 mL. The solution was diluted several times and analyzed by an atomic absorption spectrometer to determine the content of each major valent metal in the SFD.
(3) Particle size analysis. Using anhydrous ethanol as the dispersing medium and nitrogen as the carrier, the SFD after washing, drying, and sieving was subjected to particle size analysis via a Mastersizer 2000 laser particle size analyzer.
(4) Structural analysis. A Nicolet 6700 Fourier transform infrared spectrometer was used to detect and analyze the structure of the experimental raw materials, i.e., iron and steel metallurgical sintering ash, sintering ash after washing, filtrate residue after leaching, intermediate products, and final products. The instrument test conditions were as follows: resolution, 4 cm^−1^; sample prepared by a KBr press sheet; scanning, four times; and scanning range, 4000–400 cm^−1^.

#### 4.2.2. Complexing Agent Selection

**Figure 15:**
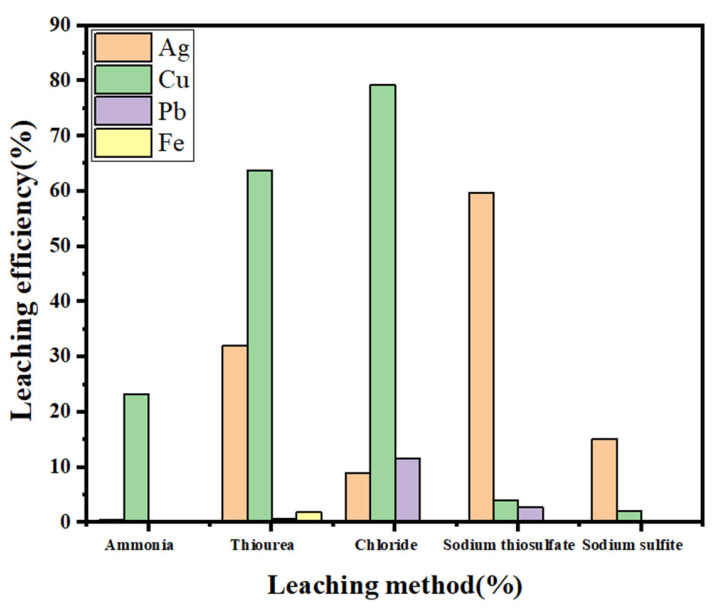
Comparison of the effects of different leaching methods.

A comparison of the complexing agents more widely used in silver recovery, such as sodium thiosulfate, thiourea, ammonia, chloride salt, and sodium sulfite, revealed that sodium thiosulfate has the highest silver leaching efficiency for SFD and that the amount of leached impurity ions is small, which can lead to selective complexation of silver; the experimental results are shown in Figure 15.

In thiosulfate leaching, ammonia and copper are added to the solution. Cu^2+^ acts as an oxidation catalyst by cycling between Cu^2+^ and Cu^+^ via redox reactions: Cu^2+^ accepts electrons from thiosulfate (reducing to Cu^+^), while dissolved oxygen regenerates Cu^2+^ by oxidizing Cu^+^. Ammonia stabilizes Cu^2+^ through complexation as Cu(NH_3_)_4_^2+^, which suppresses copper hydroxide precipitation in alkaline media and facilitates the reoxidation of Cu^+^ to Cu^2+^, thereby sustaining the catalytic cycle. The silver leaching chemistry is shown in Equations (10)–(13) [43].

Ag ⇌ Ag^+^ + e^−^ (10)

Ag^+^ + 2S_2_O_3_^2−^ ⇌ [Ag(S_2_O_3_)_2_]^3−^ (11)

Cu(NH_3_)_4_^2+^ + 3S_2_O_3_^2−^ + e^−^ ⇌ Cu(S_2_O_3_)_3_^5−^ + 4NH_3_ (12)

4Cu(S_2_O_3_)_3_^5−^ + 16NH_3_ + O_2_ + 2H_2_ O ⇌ 4Cu(NH_3_)_4_^2+^ + 4OH^−^ + 12S_2_O_3_^2−^ (13)

Equations (10)–(13) are combined to obtain the equilibrium Equation (14) for silver leaching:

4Ag + 8S_2_O_3_^2−^ + O_2_ + 2H_2_O ⇌ 4[Ag(S_2_O_3_^2−^)_2_]^3−^ + 4OH^−^ (14)

The main limitation of this system is the degradation of thiosulfate, which leads to increases in the amount of reactants used and the cost of reagents, and the degradation product (thioredoxin anion) may adversely affect the silver leaching reaction [44] and the recycling process [45]. The degradation of thiosulfate is based on Equations (15) and (16):

2Cu(NH_3_)_4_^2+^ + 8S_2_O_3_^2−^ ⇌ 2Cu(S_2_O_3_)_3_^5−^ + S_4_O_6_^2−^ + 4NH_3_ (15)

2H_2_O + 4S_2_O_3_^2−^ + O_2_ ⇌ 2S_4_O_6_^2−^ + 4OH^−^ (16)

As shown in Equations (15) and (16), copper and ammonia are not consumed in the system, but thiosulfate, which is a ligand for silver ions, is degraded over time by the tetramine complexation of copper(II) (Equation (15)) and by oxygen (Equation (16)). Thus, the amount of silver that can be leached is directly related to the amount of thiosulfate present in the solution, which is consumed in large amounts by copper(II).

According to the existing thiosulfate–copper–ammonia system leaching mechanism of silver, further analysis revealed the following: In conventional systems, cupric salts (copper(II) salts, e.g., CuCl_2_, CuSO_4_) are utilized to generate cuprous thiosulfate (Cu(S_2_O_3_)_2_^3−^) through redox reactions with thiosulfate. This process necessitates ammonia to stabilize Cu^2+^ in the solution and prevent precipitation as hydroxides (e.g., Cu(OH)_2_). However, ammonia addition introduces complexity and environmental concerns. In contrast, the modified system proposed in this study employs cuprous salts (copper(I) salts, e.g., CuCl) to directly form Cu(S_2_O_3_)_2_^3−^ without requiring prior reduction. This approach bypasses the need for ammonia, as Cu(S_2_O_3_)_2_^3−^ exhibits inherent stability in the solution and minimizes thiosulfate degradation caused by oxidative side reactions (e.g., S_2_O_3_^2−^→S_4_O_6_^2−^).

The oxidative fate of thiosulfate is governed by the applied potential. To minimize irreversible degradation, the leaching potential should be maintained within a range that stabilizes tetrathionate as the primary intermediate. This approach leverages the equilibrium between thiosulfate oxidation (S_2_O_3_^2−^→S_4_O_6_^2−^) and its regeneration from tetrathionate, thereby sustaining thiosulfate concentration in the solution. Excessive oxidation beyond tetrathionate leads to sulfate formation, which terminates the catalytic cycle.

**Figure 16:**
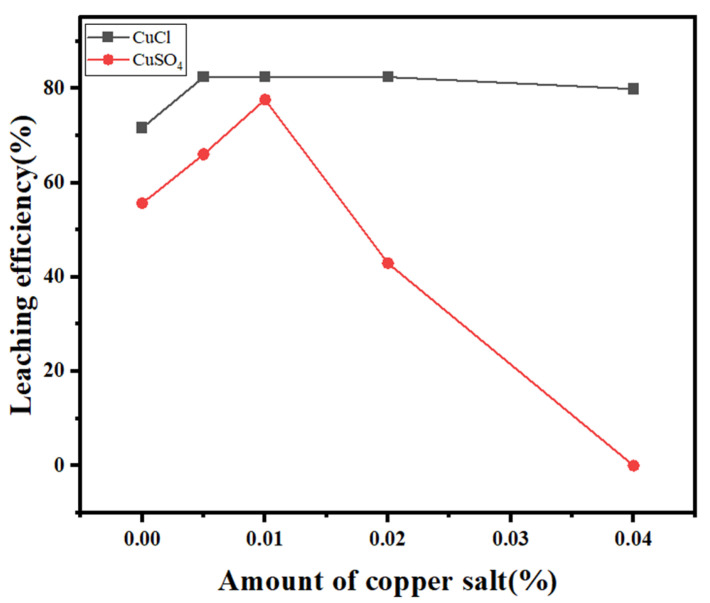
Effects of different copper salt dosages on the silver leaching efficiency.

The effects of different copper salt dosages on silver leaching in the thiosulfate leaching systems with cuprous chloride and copper sulfate under the same experimental conditions were experimentally compared, as shown in Figure 16.

As shown in the figure, when no copper salt is added, the silver leaching efficiency reaches a certain level because some copper is present in the SFD. With increasing dosage of the cuprous salt, the silver leaching efficiency reaches a peak at 0.005 mol/L of 81%. With increasing copper salt dosage, the leaching efficiency reaches its peak at 0.1 mol/L, but its value is slightly lower than that obtained with the cuprous salt. With a further increase in the dosage, the cuprous salt can maintain a high leaching efficiency, whereas the copper salt reacts with thiosulfate to generate copper sulfide due to the lack of a stabilizer and synergistically promotes the precipitation of silver, and the silver leaching efficiency decreases. The experiments reveal that the cuprous chloride–thiosulfate system not only has a better leaching effect than the copper salt–thiosulfate system but also maintains better stability without the addition of stabilizers. Therefore, cuprous chloride was finally selected as a complexing aid to determine the applicability of the Na_2_S_2_O_3_-CuCl system to silver leaching.

#### 4.2.3. Experimental Method of Complexation Leaching via the Sodium Thiosulfate Method

**Figure 17:**
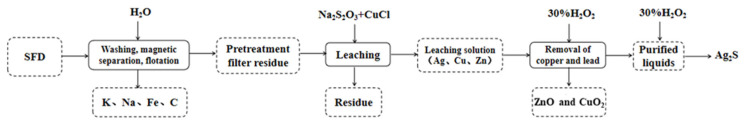
Process flow diagram for silver recovery from SFD.

The flow diagram of this process is shown in Figure 17.

## 5. Conclusions

(1) The leaching effects of different complexing agents and complexing auxiliaries on each metal element in sintered ash were compared through experiments, the possible reasons for the large difference in the silver leaching rates caused by different methods were analyzed and explored, and the Na_2_S_2_O_3_-CuCl leaching system was finally determined.
(2) Through one-factor experimental exploration and optimization of the Na_2_S_2_O_3_-CuCl co-complexation method, the recommended leaching conditions were obtained as follows: a reaction time of 120 min, a reaction temperature of 60 °C, an L/S of 6:1, a Na_2_S_2_O_3_ concentration of 45 g/L, and a CuCl dosage of 5.0 mol/L. The silver leaching rate under these conditions reached 84.3%, and the main impurity in the sintered ash was copper metal. The leaching rate of lead from the sintered ash was less than 5%, and iron was not leached, resulting in selective leaching of silver.
(3) The optimum conditions for the removal of impurity ions were determined to be as follows: a reaction time of 1 min and a 30% H_2_O_2_ dosage of 1.5 mL/100 mL of solution. Under these conditions, the conversion rate of the impurity ion lead reached 100%, the copper conversion rate reached 95%, and the silver loss rate was within 4%. The optimum recovery conditions were as follows: a reaction time of 20 min and a 30% H_2_O_2_ dosage of 0.6 mL/50 mL of solution. Under these conditions, silver at a low concentration could be completely converted, and the conversion rate of the remaining copper in the solution was less than 5%. The product obtained by filtration was silver sulfide, the purity of which was determined by titration to be approximately 97%.
(4) The leaching kinetics of the Na_2_S_2_O_3_-CuCl system and the mechanism of the removal of copper and lead impurities from the leaching solution were investigated. The removal process was divided into three steps: first, the generation of hydroxyl radicals from H_2_O_2_ catalyzed by cuprous thiosulfate; second, the reaction of hydroxyl radicals with hydrogen peroxide to generate superoxide radicals; and third, the combination of superoxide radicals with cuprous thiosulfate to generate insoluble intermediates of ternary metal ion–peroxo structure–ligand complexes.
(5) The process is easy to perform, is environmentally friendly, and significantly improves the silver recovery efficiency, providing a new technological pathway for the recovery of silver from industrial wastes, which is highly important for realizing the sustainable use of resources.

## Figures and Tables

**Table 1:** Analytical results of the contents of several elements in sintering dust (%).

Element	K	Sodium Carbonate (Chemistry)	Fe	Pb	Cu	Ag
Raw ash content (%)	18.11	3.2	16.33	1.78	0.29	160 (g/t)
Washed ash content (%)	1.3	0.6	65.3	7.1	1.2	500 (g/t)

**Table 2:** SFD characterization parameters.

Median Diameter (μm) d(0.5)	Volume Average Particle Size (μm) D[4,3]	Surface Area Average Particle Size (μm) D[3,2]	Specific Surface Area (m^2^/g)
51.1	61.1	9.7	0.6

**Table 3:** Chemical reaction and external diffusion control fitting results.

Temperature (°C)	Reaction Time (min)	Efficiency Constant k_1_	R^2^
30	30–130	1.73 × 10^−5^	0.9624
40	30–130	1.48 × 10^−5^	0.8593
50	30–130	2.23 × 10^−5^	0.8912

**Table 4:** Internal diffusion control fitting results.

Temperature (°C)	Reaction Time (min)	Efficiency Constant k_2_	R^2^
30	30–130	4.13 × 10^−5^	0.9650
40	30–130	5.19 × 10^−5^	0.8993
50	30–130	9.45 × 10^−5^	0.9189

**Table 5:** The main experimental apparatus used in the research.

Apparatus	Specification	Manufacturer
Atomic Absorption Spectrometer	ZA3000	Hitachi High-tech Co., Ltd., Tokyo, Japan
Rotary Evaporator	RE-2000A	Shanghai Yarong Co., Ltd., Shanghai, China
Circulating water vacuum pump	SHB-Ⅲ	Zhengzhou Chengke Industry and Trade Co., Ltd., Zhengzhou, China
X-ray diffraction (XRD)	D/max 2550	Rigaku Co., Ltd., Tokyo, Japan
Inductively Coupled Plasma Optical Emission Spectrometers (ICP-OES)	Optima 3000	Perkin-Elmer Co., Ltd., Waltham, MA, USA
Laser Particle Sizer	Mastersizer 2000	Malvern Instruments Co., Ltd., Malvern, UK.
Electric Blast Dryer	101–2AB	Tianjin Teste Instruments Co., Ltd., Tianjin, China
Infrared spectrometer	Nicolet 6700	Thermo Nicolet Co., Ltd., Madison, WI, USA

**Table 6:** The main experimental reagents used in the research.

Reagent	Specification	Manufacturer
Sodium dodecylbenzene sulfonate	AR	Sinopharm Chemical Reagent Co., Ltd., Shanghai, China
Sodium hyposulfide	AR	Hunan Huihong Reagent Co., Ltd., Changsha, China
Sodium sulfurous acid	AR	Hunan Huihong Reagent Co., Ltd., Changsha, China
Cuprous chloride	AR	Shanghai Maclean Reagent Co., Ltd., Shanghai, China
Ammonia	AR	Sinopharm Chemical Reagent Co., Ltd., Shanghai, China
30% Hydrogen peroxide	AR	Hunan Huihong Reagent Co., Ltd., Changsha, China
Silver nitefficiency	AR	Guangzhou Jinzhujiang Chemical Co., Ltd., Guangzhou, China

## Data Availability

The original contributions presented in this study are included in the article material. Further inquiries can be directed to the corresponding authors.
